# Novel ZNFX1 Variants Associated With Refractory Anemia and Interstitial Lung Disease

**DOI:** 10.1002/mgg3.70329

**Published:** 2026-11-10

**Authors:** Lu Jiang, Chan Huang, Xiaohan Hu, Xueqian Wang, Jiawei Chen, Canhong Zhu, Xiaozhong Li, Aiqing Zhang

**Affiliations:** ^1^ Department of Nephrology and Immunology Children's Hospital of Soochow University Suzhou China; ^2^ Institute of Pediatrics, Children's Hospital of Soochow University Suzhou China; ^3^ Medical Genetics Laboratory Children's Hospital of Soochow University Suzhou China; ^4^ Department of Respiratory Medicine Children's Hospital of Soochow University Suzhou China

**Keywords:** genotype–phenotype correlation, interferon‐stimulated genes, interstitial lung disease, whole‐exome sequencing, ZNFX1 deficiency

## Abstract

**Background:**

ZNFX1 deficiency is a rare inborn error of immunity characterized by recurrent infections, hyperinflammation, cytopenias, and multiorgan involvement. Owing to the limited number of reported cases, its clinical and molecular spectrum remains incompletely defined.

**Methods:**

Clinical evaluation, whole‐exome sequencing (WES) of the proband, Sanger sequencing for variant validation and segregation analysis, RNA sequencing, AlphaFold‐based structural modeling, and interferon‐stimulated gene (ISG) expression analysis were performed. Variants were interpreted according to the American College of Medical Genetics and Genomics (ACMG) guidelines.

**Results:**

A Chinese girl presented with refractory anemia, hyperinflammation, digital clubbing, developmental delay, and interstitial lung disease (ILD). WES of the proband identified three previously unreported ZNFX1 variants. Subsequent Sanger sequencing and segregation analysis confirmed that c.981dupA (p.Asp328Argfs*4) was paternally inherited, whereas c.1605_1630delinsGGGAATATCGTGGA (p.Asn535_Val543delinsLysGlyIleSerTrp) and c.2893G>A (p.Glu965Lys) were maternally inherited. According to ACMG criteria, one variant was classified as likely pathogenic and the other two as variants of uncertain significance. RNA sequencing demonstrated reduced ZNFX1 expression, while AlphaFold‐based modeling suggested local structural alterations. ISG expression analysis indicated activation of Type I interferon signaling.

**Conclusion:**

We report a child with ZNFX1 deficiency carrying three previously unreported ZNFX1 variants associated with refractory anemia and ILD. These findings expand both the molecular and phenotypic spectrum of ZNFX1 deficiency and highlight the value of integrating genomic, transcriptomic, structural modeling, and ISG expression analyses for molecular diagnosis.

## Graphical abstract

We identified three novel ZNFX1 variants in a child with refractory anemia and interstitial lung disease. Integrated transcriptomic, structural, and selected interferon‐stimulated gene expression analyses supported the molecular diagnosis and expanded the mutational and clinical spectrum of ZNFX1 deficiency, highlighting associated immune dysregulation.

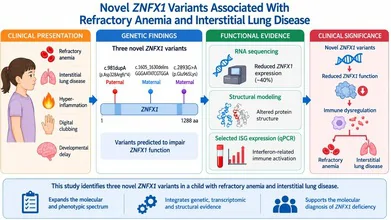

## 1 Introduction

Zinc finger NFX1‐type containing 1 (ZNFX1) is an interferon‐stimulated gene (ISG) that encodes a highly conserved double‐stranded RNA (dsRNA) sensor involved in innate antiviral immunity. ZNFX1 recognizes viral RNA and interacts with mitochondrial antiviral signaling protein (MAVS), thereby promoting downstream interferon signaling and antiviral responses (Cheng and Parker 2025). Experimental studies have further demonstrated that ZNFX1 contributes to the restriction of viral replication and the maintenance of immune homeostasis (Wang et al. 2019).

ZNFX1 deficiency is a rare autosomal recessive inborn error of immunity that typically presents during infancy or early childhood. Since the first description of this disorder, only a limited number of affected individuals have been reported worldwide. Clinical manifestations are highly heterogeneous and include recurrent infections, cytopenias, hyperinflammation, hepatosplenomegaly, lymphadenopathy, neurological abnormalities, and progressive multiorgan involvement. Interstitial lung disease (ILD) and hemophagocytic lymphohistiocytosis (HLH)‐like episodes have also been observed in a subset of patients, highlighting the broad clinical spectrum associated with this condition (Al‐Saud et al. 2023; Alawbathani et al. 2022; Le Voyer et al. 2021; Vavassori et al. 2021).

Despite increasing recognition of ZNFX1 deficiency, its clinical and molecular spectrum remains incompletely characterized, and genotype–phenotype correlations are poorly understood because of the limited number of reported cases. Here, we report a Chinese patient with ZNFX1 deficiency carrying three previously unreported variants. Through comprehensive clinical and genetic characterization, together with a review of previously reported cases, we further expand the mutational and phenotypic spectrum of this disorder and provide additional insights into its clinical manifestations.

## 2 Materials and Methods

### 2.1 Subjects and Ethics Approval

The study was approved by the Ethics Committee of the Children's Hospital of Soochow University (approval number: 2025CS199). Written informed consent was obtained from the patient's parents for participation in the study and publication of relevant clinical data. All procedures were performed in accordance with the Declaration of Helsinki.

### 2.2 Genetic Analysis

Genomic DNA was extracted from peripheral blood using the HiPure Tissue & Blood DNA Kit (Magen, D3018). DNA quality and concentration were evaluated using a NanoDrop 2000 spectrophotometer and Qubit 2.0 Fluorometer. Whole‐exome sequencing (WES) was performed to identify potential disease‐causing variants. Sequencing reads were aligned to the human reference genome (GRCh38/hg38) using Burrows–Wheeler Aligner. PCR duplicates were marked with Picard tools. Single nucleotide variants and small insertions or deletions were identified using the Sentieon DNAseq pipeline, while copy number variants were analyzed with ExomeDepth. Variants were annotated using ANNOVAR. Population allele frequencies were evaluated using the Genome Aggregation Database (gnomAD v4.1.1). Previously reported disease‐associated variants were evaluated using the Human Gene Mutation Database (HGMD Professional 2025.2) and ClinVar. Variants with a minor allele frequency (MAF) < 0.01 in gnomAD were prioritized for further analysis. Variant classification was performed according to the American College of Medical Genetics and Genomics/Association for Molecular Pathology (ACMG) guidelines.

### 2.3 RNA Sequencing Analysis

Total RNA was extracted from peripheral whole blood samples for library preparation and paired‐end sequencing. Sequencing quality was assessed using standard metrics, including read quality scores and alignment rate. After quality control, reads were aligned to the human reference genome (GRCh38/hg38). Gene expression outliers were identified using the OUTRIDER algorithm.

### 2.4 Protein Structure Prediction

Three‐dimensional structures of wild‐type ZNFX1 and its variants were predicted using AlphaFold 3 (Abramson et al. 2024) via the AlphaFold Server (https://alphafoldserver.com/). The wild‐type human ZNFX1 protein sequence was obtained from UniProt (accession number Q9P2E3, canonical isoform, 1918 amino acids). Variant sequences were generated by introducing the corresponding amino acid changes into the reference sequence. Wild‐type and variant sequences were submitted separately for structure prediction using identical default server settings. For each sequence, the highest‐ranked model, based on the server‐provided ranking score, was selected for subsequent analysis. No additional Amber relaxation was performed on the server‐generated models. Structural visualization and hydrogen‐bond analysis were conducted using UCSF ChimeraX version 1.5 (Pettersen et al. 2021). Putative hydrogen bonds were identified using the default geometric criteria.

### 2.5 PBMC Isolation and ISG Expression Analysis

Peripheral blood mononuclear cells (PBMCs) were isolated from fresh whole blood by Ficoll density‐gradient centrifugation. Total RNA was extracted and reverse‐transcribed into complementary DNA (cDNA). The expression levels of a panel of ISGs were measured by quantitative real‐time PCR to assess Type I interferon pathway activation. GAPDH was used as the internal reference gene, and relative gene expression was calculated using the 2^−ΔΔCt^ method. Primer sequences are provided in Table S1.

### 2.6 Proinflammatory Cytokine Analysis

Serum levels of IL‐6, IFN‐γ, TNF‐α, IL‐1RA, and CD163 were measured using a Luminex‐based multiplex assay. Briefly, serum samples were analyzed on a Luminex 200 system (Luminex Trading [Shanghai] Co. Ltd., Shanghai, China) using the LZ2R‐CAL‐K25 calibration kit and LX2R‐PVER‐K25 assay kit according to the manufacturer's instructions. Analyte concentrations were calculated from standard curves generated using the corresponding calibration standards. Values exceeding the upper limits of the laboratory‐specific reference intervals were considered elevated.

## 3 Results

### 3.1 Clinical Phenotype

The patient was a 1‐year‐old Chinese girl born to healthy non‐consanguineous parents. She was first admitted at 9 months of age because of recurrent anemia that had persisted for 3 months. Laboratory evaluation showed normocytic anemia with persistently elevated inflammatory markers. Investigations for nutritional deficiencies, hemolysis, common viral infections, and bone marrow abnormalities were unremarkable.

On subsequent evaluation, she exhibited marked digital clubbing (Figure 1A) and developmental delay. Laboratory studies demonstrated persistent systemic inflammation, with elevated levels of C‐reactive protein, erythrocyte sedimentation rate, immunoglobulin G, and ferritin. Brain magnetic resonance imaging revealed delayed myelination of the bilateral periventricular white matter (Figure 1B). High‐resolution computed tomography (HRCT) of the chest demonstrated multiple patchy pulmonary opacities, bilateral pleural thickening, and numerous cystic lucencies (Figure 1C). Serum KL‐6 levels were markedly elevated (> 10,000 U/mL; reference range < 500 U/mL), supporting a diagnosis of interstitial lung disease (ILD). Further immunological evaluation revealed elevated levels of several inflammatory mediators, including IL‐6 (58.3 pg/mL; reference value ≤ 5.1 pg/mL), IFN‐γ (37.5 pg/mL; ≤ 8.2 pg/mL), TNF‐α (6.5 pg/mL; ≤ 4.1 pg/mL), IL‐1RA (4407.2 pg/mL; ≤ 3156 pg/mL), and CD163 (2,888,399.2 pg/mL; ≤ 1,540,721 pg/mL), consistent with ongoing immune dysregulation and hyperinflammation. Overall, the patient presented with a distinctive phenotype characterized by refractory anemia, digital clubbing, hyperinflammation, developmental delay, and ILD.

**FIGURE 1:**
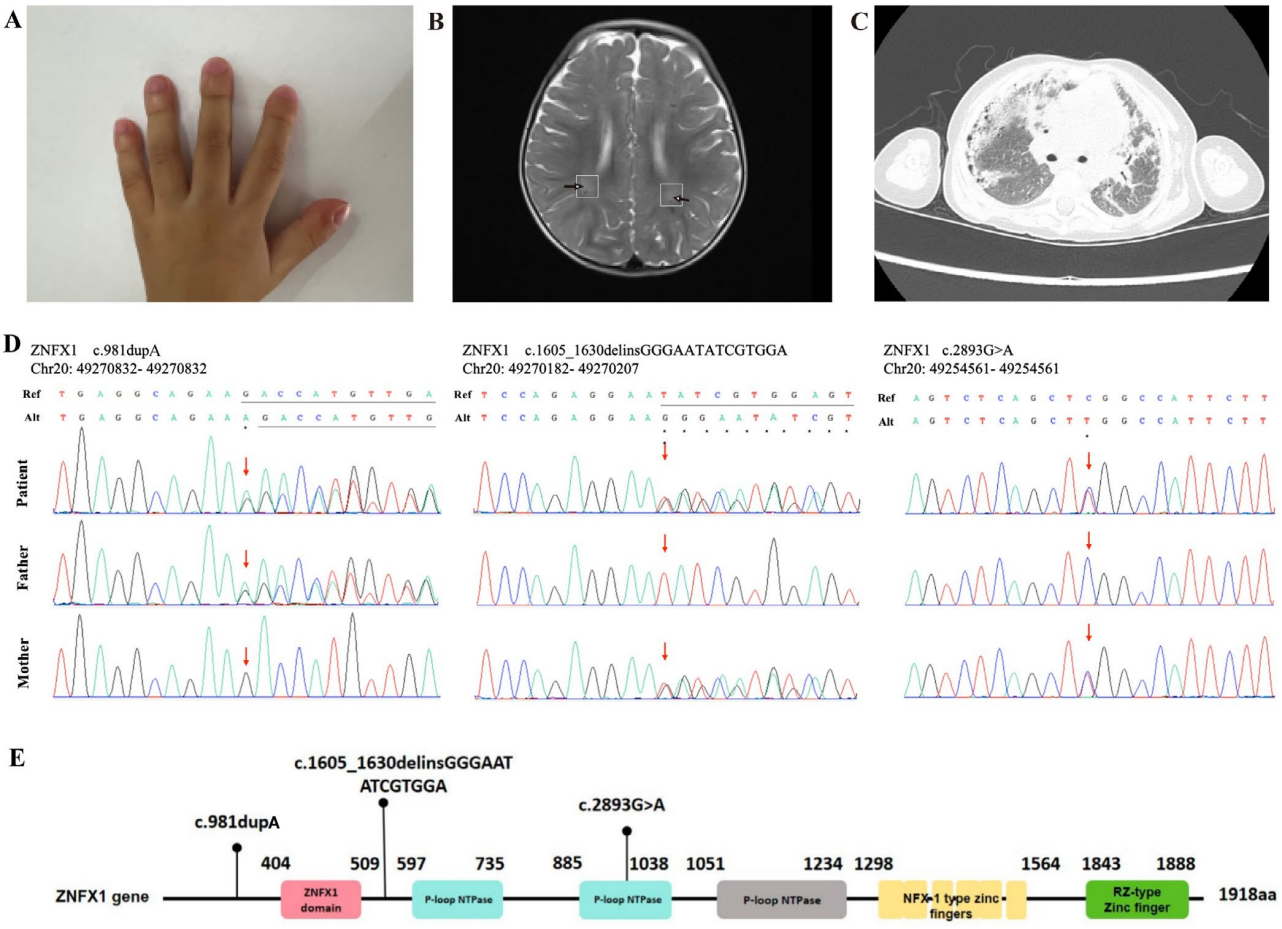
Clinical, radiological, and genetic findings in the patient with ZNFX1 variants. (A) Clinical photograph showing digital clubbing. (B) Brain magnetic resonance imaging (MRI; T2‐weighted image) demonstrating delayed myelination in the bilateral periventricular white matter (arrow). (C) High‐resolution computed tomography (HRCT) of the chest showing patchy pulmonary opacities, bilateral pleural thickening, and multiple cystic lucencies. (D) Sanger sequencing confirmed a paternally inherited c.981dupA variant and two maternally inherited variants, c.1605_1630delinsGGGAATATCGTGGA and c.2893G>A, in ZNFX1. (E) Schematic representation of the ZNFX1 protein showing the locations of the identified variants: c.981dupA, c.1605_1630delinsGGGAATATCGTGGA, and c.2893G>A.

During hospitalization, she was treated with cefoperazone–sulbactam, meropenem, methylprednisolone (2 mg/kg), intravenous immunoglobulin (1 g/kg), tocilizumab (80 mg every 2 weeks), oral sulfamethoxazole, and acetylcysteine. Following treatment, the inflammatory markers normalized, and the patient was subsequently discharged. At the 9‐month follow‐up, she remained on low‐dose prednisone (2.5 mg once daily) and monthly tocilizumab. Her anemia had resolved, digital clubbing had improved, and follow‐up HRCT showed marked resolution of the pulmonary abnormalities. Her clinical condition remained stable under maintenance therapy.

### 3.2 Genetic Analysis

WES of the proband identified three ZNFX1 variants: c.981dupA (p.Asp328Argfs*4), c.1605_1630delinsGGGAATATCGTGGA (p.Asn535_Val543delinsLysGlyIleSerTrp), and c.2893G>A (p.Glu965Lys). Subsequent Sanger sequencing of the proband and both parents confirmed these variants and their parental origin. The c.981dupA variant was inherited from the father, whereas c.1605_1630delinsGGGAATATCGTGGA and c.2893G>A were inherited from the mother (Figure 1D). The locations of the three variants within ZNFX1 are illustrated in Figure 1E. Integrative Genomics Viewer (IGV) visualization of the c.1605_1630delinsGGGAATATCGTGGA variant is provided in Figure S1. Extended Sanger sequencing chromatograms for this variant are shown in Figure S2.

The c.981dupA and c.1605_1630delinsGGGAATATCGTGGA variants were absent from the gnomAD v4.1.1 exome dataset. The c.2893G>A missense variant was rare, with an overall allele frequency of 8.89 × 10^−6^ and an East Asian allele frequency of 1.76 × 10^−4^. Several in silico prediction tools suggested a potentially deleterious effect of this variant (Table 1). According to ACMG guidelines, c.981dupA was classified as likely pathogenic, whereas c.1605_1630delinsGGGAATATCGTGGA and c.2893G>A were classified as variants of uncertain significance (VUS).

**TABLE 1:** Genetic characteristics of the ZNFX1 variants identified in the patient.

Variant	c.981dupA	c.1605_1630delinsGGGAATATCGTGGA	c.2893G>A
Genome assembly	GRCh38	GRCh38	GRCh38
Reference transcript	NM_021035.3	NM_021035.3	NM_021035.3
Genomic coordinate	NC_000020.11: g.49270832dup	NC_000020.11:g.49270182_49270207 delinsTCCACGATATTCCC	NC_000020.11: g.49254561C>T
Variant type	Frameshift	In‐frame deletion–insertion	Missense
Protein change	p.Asp328Argfs*4	p.Asn535_Val543delinsLysGlyIleSerTrp	p.Glu965Lys
Population frequency (gnomAD)	Not detected	Not detected	Overall AF: 8.89 × 10^−6^ East Asian AF: 1.76 × 10^−4^
CADD PHRED score	N/A	N/A	25.3
SIFT	N/A	N/A	0.02 (Deleterious)
PolyPhen‐2	N/A	N/A	HDIV: 0.983 (Probably damaging) HVAR: 0.753 (Possibly damaging)
PROVEAN	N/A	N/A	−3.17 (Deleterious)
MutationTaster	N/A	N/A	1.0 (Disease causing)
REVEL	N/A	N/A	0.401
ACMG classification	Likely pathogenic	VUS	VUS

Abbreviations: AF, allele frequency; N/A, not applicable; VUS, variant of uncertain significance.

### 3.3 RNA Sequencing Analysis and Protein Structural Modeling

RNA sequencing analysis using the OUTRIDER algorithm (Brechtmann et al. 2018) demonstrated an approximately 40% reduction in ZNFX1 expression compared with controls (Figure 2A). Because c.981dupA is predicted to introduce a premature termination codon, the reduced transcript abundance is consistent with nonsense‐mediated mRNA decay and provides functional support for the likely pathogenic effect of this truncating variant. Structural modeling using AlphaFold 3 further suggested that the two non‐truncating variants, c.1605_1630delinsGGGAATATCGTGGA and c.2893G>A, may affect the structural stability of ZNFX1 (Figure 2B,C). For c.1605_1630delinsGGGAATATCGTGGA, structural comparison revealed an altered spatial configuration of residues within the affected region (Asn535‐Val543), accompanied by the loss of multiple hydrogen bonds, suggesting reduced local structural stability (Figure 2B). Similarly, the c.2893G>A variant resulted in altered local molecular interactions at Glu965, disruption of hydrogen‐bond networks, and conformational changes in the surrounding region (Figure 2C). Collectively, these findings suggest that the two non‐truncating variants may affect ZNFX1 protein stability and provide additional support for their potential functional relevance.

**FIGURE 2:**
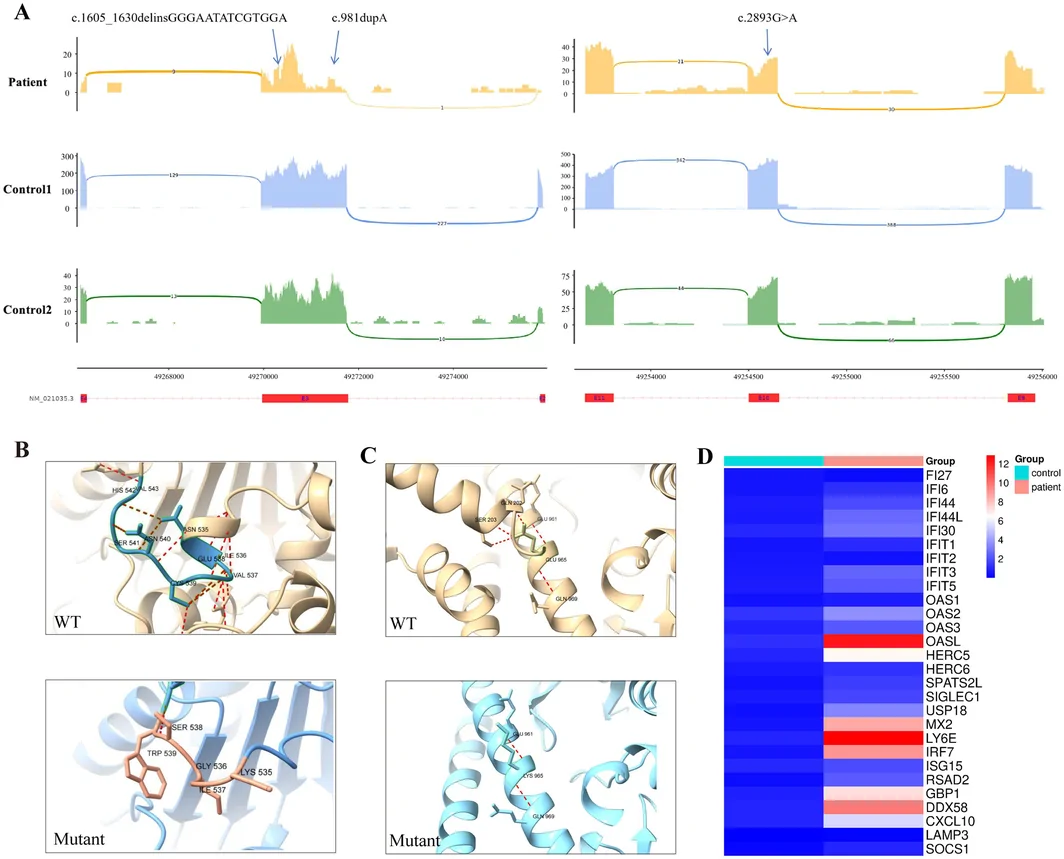
Functional characterization of the identified ZNFX1 variants and interferon pathway activation. (A) RNA sequencing analysis showing reduced ZNFX1 expression in the patient compared with controls. Quantitative analysis using the OUTRIDER algorithm demonstrated an approximately 40% reduction in transcript expression. Variant positions are indicated. (B) AlphaFold‐predicted structures of wild‐type and mutant ZNFX1 carrying the c.1605_1630delinsGGGAATATCGTGGA variant. Residues within the affected region (Asn535‐Val543) are labeled. Dashed lines indicate hydrogen‐bond interactions. (C) AlphaFold‐predicted structures of wild‐type and mutant ZNFX1 carrying the c.2893G>A (p.Glu965Lys) variant. The affected residue is labeled, and dashed lines indicate local molecular interactions. (D) Heat map showing expression of interferon‐stimulated genes (ISGs) in peripheral blood mononuclear cells from the patient and healthy controls. Genes are grouped according to functional categories. Color intensity represents relative gene expression levels.

### 3.4 Analysis of Type I Interferon‐Stimulated Gene Expression

To further characterize the patient's inflammatory profile, quantitative real‐time PCR was performed to assess the expression of selected ISGs in PBMCs from the patient and a healthy control. Genes were organized according to functional categories in the heat map to facilitate visualization. Compared with the healthy control, the patient exhibited increased expression of several ISGs, including OASL, MX2, LY6E, IRF7, DDX58, and GBP1 (Figure 2D), suggesting activation of the Type I interferon signaling pathway. These findings support enhanced interferon‐related immune activity in the patient.

## 4 Discussion

We describe a Chinese child with ZNFX1 deficiency characterized by refractory anemia, hyperinflammation, digital clubbing, developmental delay, and ILD, carrying three previously unreported ZNFX1 variants.

Previous reports have described a broad clinical spectrum of ZNFX1 deficiency, including hyperinflammatory or HLH‐like episodes, hematologic abnormalities, neurological manifestations, and pulmonary involvement (Al‐Saud et al. 2023; Alawbathani et al. 2022; Cheng and Parker 2025; Lohaibi et al. 2026). Pulmonary involvement, particularly ILD, appears to be an important but underrecognized feature of ZNFX1 deficiency. Our patient developed severe ILD characterized by digital clubbing, diffuse radiographic abnormalities, and markedly elevated serum KL‐6 levels. Similar pulmonary manifestations have been reported in only a limited number of affected patients, suggesting that lung involvement may represent a clinically relevant component of the phenotype (Al‐Saud et al. 2023). Notably, dysregulated Type I interferon signaling has also been implicated in the pathogenesis of ILD in several autoimmune and autoinflammatory disorders (Junfei et al. 2026; Piera‐Velazquez et al. 2021; Zhang et al. 2019).

ZNFX1 deficiency is currently recognized as a rare inborn error of immunity characterized by susceptibility to severe viral infections and early‐onset multisystem inflammation (Poli et al. 2025; Vavassori et al. 2021). Patients with ZNFX1 deficiency have also been included among rare interferonopathy conditions in a recent cohort of monogenic interferon‐mediated diseases (Al‐Saleem et al. 2024). Type I IFN signalling upregulation has been observed in ZNFX1‐associated disease; however, Crow's expanded classification dataset places ZNFX1 within the subgroup “Uncertain link to enhanced Type I interferon signalling” (Crow 2024). Thus, ZNFX1 deficiency shows clinical and immunological overlap with Type I interferonopathies, but its position within this disease spectrum remains incompletely defined.

This distinction is particularly relevant to the apparent immunological paradox of ZNFX1 deficiency. Type I IFNs are essential for antiviral defense, whereas persistent enhancement of Type I IFN signalling can drive autoinflammatory and autoimmune disease and is a defining feature of Type I interferonopathies (Crow and Casanova 2024). Most established Type I interferonopathies are not primarily characterized by severe viral susceptibility. In contrast, ZNFX1 deficiency can combine severe viral infections with increased ISG expression and systemic inflammation. Experimental findings provide a biological basis for this coexistence. Vavassori et al. reported increased baseline ISG expression and dysregulated responses to intracellular nucleic acid stimulation in ZNFX1‐deficient patients, while monocytes from an affected patient showed impaired viral clearance following poly(I:C) stimulation (Vavassori et al. 2021). A recent study has shown that ZNFX1 suppresses NLRP3 inflammasome activation, providing a mechanistic link between ZNFX1 dysfunction and excessive inflammation (Huang et al. 2024). ZNFX1 can directly restrict RNA virus replication through viral RNA sequestration, and disease‐associated variants can impair this antiviral activity (Squair et al. 2026). Importantly, this antiviral restriction can operate in parallel to interferon signalling. Together, these observations help explain how impaired viral control and hyperinflammation can coexist in ZNFX1 deficiency. In our patient, increased expression of selected ISGs was consistent with the interferon‐related immune activation reported in this disorder. However, further studies incorporating standardized interferon scoring and additional validation are needed to determine whether these findings reflect a bona fide Type I interferon signature.

## 5 Conclusion

Our findings expand the mutational and clinical spectrum of ZNFX1 deficiency and further highlight the immune dysregulation associated with this disorder. Integration of clinical, genetic, transcriptomic, structural, and ISG expression data provided additional support for variant interpretation and molecular characterization. These findings may improve the recognition and genetic evaluation of patients with unexplained multisystem inflammation and pulmonary involvement.

## Author Contributions

**Lu Jiang:** conceptualization, methodology, investigation, data curation, formal analysis, visualization, writing – original draft, writing – review and editing. **Chan Huang:** methodology, investigation, data curation, formal analysis, writing – original draft. **Xiaohan Hu:** formal analysis, data curation. **Xueqian Wang:** visualization, validation. **Jiawei Chen:** investigation. **Canhong Zhu:** supervision, writing – original draft. **Xiaozhong Li:** investigation, conceptualization. **Aiqing Zhang:** conceptualization, methodology, supervision, writing – review and editing.

## Funding

This work was supported by grants from Suzhou Science and Technology Plan Project (Grant No. SYW2025119) and the Key Project of Science, Education, and Health Strengthening in Suzhou (Grant No. ZDXM2025009).

## Ethics Statement

This study was approved by the Ethics Committee of Children's Hospital of Soochow University (2025CS199).

## Consent

Written informed consent for publication was obtained from the patient's parents.

## Conflicts of Interest

The authors declare no conflicts of interest.

## Supporting information


**Figure S1:** Integrative Genomics Viewer (IGV) visualization of the ZNFX1 c.1605_1630delinsGGGAATATCGTGGA variant in the patient.
**Figure S2:** Extended Sanger sequencing chromatograms of the ZNFX1 c.1605_1630delinsGGGAATATCGTGGA variant in the patient and her parents.

**Table S1:** Primer sequences.

## Data Availability

The data that support the findings of this study are available on request from the corresponding author. The data are not publicly available due to privacy or ethical restrictions.
