# Supplementary material for: Novel ZNFX1 Variants Associated With Refractory Anemia and Interstitial Lung Disease
Source: Mol Genet Genomic Med. 2026 Nov 10;14(11):e70329. doi: 10.1002/mgg3.70329 (PMC13750346; doi:10.1002/mgg3.70329)
Supplement: Supplementary file 1 [file MGG3-14-e70329-s001.docx]

Supplementary Table 1: Primer sequences

| Gene name | 5’- 3’ sequence |
| --- | --- |
| DDX58 | F: CACCTCAGTTGCTGATGAAGGC  R: GTCAGAAGGAAGCACTTGCTACC |
| IFIT2 | F: GACACGGTTAAAGTGTGGAGG  R: TCCAGACGGTAGCTTGCTATT |
| CXCL10 | F: GTGGCATTCAAGGAGTACCTC  R: TGATGGCCTTCGATTCTGGATT |
| GBP1 | F: AGGAGTTCCTTCAAAGATGTGGA  R: GCAACTGGACCCTGTCGTT |
| LAMP3 | F: TGGGAGCCTATTTGACCGTCTC  R: GCTGACAACTGGAGGCTCTGTT |
| IFIT5 | F: GGCCAAAATAAAGACGCCCTT  R: GACCAGGCTTCGTACTTCTTC |
| IFI27 | F: TGCTCTCACCTCATCAGCAGT  R: CACAACTCCTCCAATCACAACT |
| IFI44L | F: AGCCGTCAGGGATGTACTATAAC  R: AGGGAATCATTTGGCTCTGTAGA |
| HERC5 | F: CAACTGGGAGAGCCTTGTGGTT  R: CTGGACCAGTTTGCTGAAAGTGG |
| IFI44 | F: GTGAGGTCTGTTTTCCAAGGGC  R: CGGCAGGTATTTGCCATCTTTCC |
| RSAD2 | F: CCAGTGCAACTACAAATGCGGC  R: CGGTCTTGAAGAAATGGCTCTCC |
| OAS2 | F: GCTTCCGACAATCAACAGCCAAG  R: CTTGACGATTTTGTGCCGCTCG |
| OAS3 | F: GAAGGAGTTCGTAGAGAAGGCG  R: CCCTTGACAGTTTTCAGCACC |
| SIGLEC1 | F: ACCTGGAGGAAACTGACAGTGG  R: CTCAGTGTCACTGCCTGTCCTT |
| IFIT1 | F: GCGCTGGGTATGCGATCTC  R: CAGCCTGCCTTAGGGGAAG |
| OAS1 | F: TGTCCAAGGTGGTAAAGGGTG  R: CCGGCGATTTAACTGATCCTG |
| SOCS1 | F: TTCGCCCTTAGCGTGAAGATGG  R: TAGTGCTCCAGCAGCTCGAAGA |
| IRF7 | F: CCCACGCTATACCATCTACCT  R: GATGTCGTCATAGAGGCTGTTG |
| LY6E | F: GACCAGGACAACTACTGCGTGA  R: AAGCCACACCAACATTGACGCC |
| USP18 | F: CCTGAGGCAAATCTGTCAGTC  R: CGAACACCTGAATCAAGGAGTTA |
| ISG15 | F: TGGACAAATGCGACGAACCTC  R: TCAGCCGTACCTCGTAGGTG |
| OASL | F: CCATTGTGCCTGCCTACAGAG  R: CTTCAGCTTAGTTGGCCGATG |
| IFI30 | F: CCCCTCTGCAAGCGTTAGAC  R: CCCGCAGGTATAGATTGCCT |
| IFI6 | F: CTCTTCACTTGCAGTGGGGT  R: ATTCAGGATCGCAGACCAGC |
| SPATS2L | F: CTGACTGCTCGTCAGAAGAAAGC  R: CTCCTCGTCATATTTACGCTCGC |
| MX2 | F: AAAAGCAGCCCTGTGAGGCATG  R: GTGATCTCCAGGCTGATGAGCT |
| HERC6 | F: GGAGAAACGACTTCCATTGATGTG  R: AGCTCTGAGCAGATCATCCTCG |
| IFIT3 | F: AAAAGCCCAACAACCCAGAAT  R: CGTATTGGTTATCAGGACTCAGC |
| GAPDH | F: GGAGCGAGATCCCTCCAAAAT  R: GGCTGTTGTCATACTTCTCATGG |
