# Supplementary material for: Novel ZNFX1 Variants Associated With Refractory Anemia and Interstitial Lung Disease
Source: Mol Genet Genomic Med. 2026 Nov 10;14(11):e70329. doi: 10.1002/mgg3.70329 (PMC13750346; doi:10.1002/mgg3.70329)
Supplement: Supplementary file 2 [file MGG3-14-e70329-s002.docx]

**Supplementary Figure 1 | Integrative Genomics Viewer (IGV) visualization of the ZNFX1 c.1605_1630delinsGGGAATATCGTGGA variant in the patient.**

The IGV screenshot shows sequencing coverage and aligned reads at the variant locus. A total of 66 reads covered the locus, with 32 reads supporting the complex deletion-insertion event, consistent with heterozygosity. Coordinates are based on the GRCh38 reference genome.

**Supplementary Figure 2 | Extended Sanger sequencing chromatograms of the ZNFX1 c.1605_1630delinsGGGAATATCGTGGA variant in the patient and her parents.**

The chromatograms were extended beyond the breakpoint to allow clearer visualization of the downstream sequence and peak patterns. The patient and her mother showed overlapping peaks downstream of the variant site, whereas the father showed the wild-type sequence, consistent with maternal inheritance of the variant. Red arrows indicate the variant site.
